# Multiple dietary fiber for gestational hyperinsulinism: A protocol of systematic review and meta-analysis of randomized clinical trials

**DOI:** 10.1097/MD.0000000000022266

**Published:** 2020-10-02

**Authors:** Botong Yang, Mengzhu Wu, Xinxia Zhang, Jia Xia, Lizhen Wang, QI Chen, Min Zhong, Xiaoming Tang

**Affiliations:** a Hospital of Chengdu University of Traditional Chinese Medicine; b Chengdu University of Traditional Chinese Medicine, Chengdu, Sichuan, China.

**Keywords:** diet, hyper insulinemia in pregnancy, meta-analysis and systematic review, multiple dietary fiber, protocol

## Abstract

**Introduction:**

Gestational hyperinsulinism is a metabolic disease which is widely concerned at home and abroad. It is a clinical consensus that the embryo implantation ability of patients with hyperinsulinemia is decreased and the abortion rate after implantation is high. The treatment of gestational hyperinsulinism with Multiple dietary fiber diets has been proven. However, due to the lack of evidence, there is no specific method or recommendation, it is necessary to carry out a systematic evaluation of Multiple dietary fiber diet, to provide effective evidence for further research.

**Methods and analysis:**

The following databases will be searched from their inception to August 2020: Electronic database includes PubMed, Embase, Cochrane Library, Web of Science, Nature, Science online, Chinese Biomedical Database WanFang, VIP medicine information, and CNKI. Primary outcomes: Fasting glucose, fasting insulin, homeostasis model assessment of insulin resistance, glycosylated hemoglobin. Additional outcomes: Low Density Lipoprotein (LDL), High Density Lipoprotein (HDL), triglycerides (TG), total serum cholesterol (TC). Data will be extracted by 2 researchers independently, risk of bias of the meta-analysis will be evaluated based on the Cochrane Handbook for Systematic Reviews (SR) of Interventions. All data analysis will be conducted by data statistics software Review Manager V.5.3. and Stata V.12.0.

**Results:**

The results of this study will systematically evaluate the effectiveness and safety of Multiple dietary fiber diet interventions in the treatment of gestational hyperinsulinism.

**Conclusion:**

The SR of this study will summarize the current published evidence of Multiple dietary fiber for the treatment of gestational hyperinsulinism, which can further guide the promotion and application of it.

**Ethics and dissemination:**

This study is a SR, the outcomes are based on the published evidence, so examination and agreement by the ethics committee are not required in this study. We intend to publish the study results in a journal or conference presentations.

**Open Science Fra network (OSF) registration number:**

August 19, 2020. osf.io/tbc7z. (https://osf.io/tbc7z)

## 1 Introduction

With social progress and changes of lifestyle, the quantity of patients with obesity, polycystic ovary syndrome and other diseases is gradually increasing, and these diseases often lead to hyperinsulinemia and insulin resistance.[^1^,^2^,^3^] At present, studies on the effect of insulin mainly focus on metabolism, but clinical observation also found that in some patients with diseases characterized by hyperinsulinemia, the ability of embryo implantation is reduced and the rate of abortion after implantation is high.[^4^,^5^] If pre-pregnancy patients have hyperinsulinemia, the level of insulin resistance of pregnant women increases with the increase of gestational age, insulin sensitivity decreases, and hyperinsulinemia and insulin resistance are generally aggravated.^[6]^ Therefore, for pregnant women whose blood glucose has not reached the level of insulin use, how to safely and effectively improve hyperinsulinemia is needed for clinical research.

In recent years, the control of blood glucose through dietary intervention has become a research hotspot. Good dietary management is conducive to the standardization of blood glucose and the reduction of blood glucose fluctuation.^[7]^ Moreover, dietary fiber therapy has the advantages of high safety and compliance. Multiple dietary fiber has a relatively clear effect on the improvement of insulin resistance in type 2 diabetes,^[8]^ but there is still a lack of evaluation of the efficacy and safety of multiple dietary fibers for insulin levels during pregnancy. To develop and promote Vigorously the improvement effect of diet on gestational hyperinsulinism and to improve to give a good birth and good care are clinical requirements. Therefore, this study intends to use the systematic evaluation and meta-analysis method of multiple dietary fiber in the treatment of gestational hyperinsulinism to evaluate its efficacy and safety.

## 2 Methods

### 2.1 Study registration

The protocol has been registered in OSF (Open Science Fra network) Preregistration. August 19, 2020. osf.io/tbc7z. (https://osf.io/tbc7z). The protocol will follow the statement guidelines of Preferred Reporting Items for Systematic Reviews and Meta-Analyses Protocols (PRISMAP),^[9]^ Changes will be reported in the full review as required.

### 2.2 Inclusion and exclusion criteria for study selection

#### 2.2.1 Inclusion criteria

Inclusion criteria are all randomized controlled trials (RCTS), which multiple dietary fibers are used to treat gestational hyperinsulinism. The language of the trials to be included only Chinese or English.

#### 2.2.2 Exclusion criteria. Following studies will be excluded

1. patients age <18 years old
2. There are type 2, and special types of diabetes before pregnancy.
3. the treatment was combined with other therapies other than lifestyle.
4. Non-RCTs and Quasi-RCTs
5. Case series and Reviews
6. Animal studies

### 2.3 Types of participants

Types of participants included people diagnosed with gestational hyperinsulinism, no matter the degree and possible complications. All the patients should be treated by Multiple dietary fiber with other conventional treatments. No sex, ethnicity, or education restrictions is here.

### 2.4 Experimental interventions

Multiple dietary fiber should be the main treatments which Contains recipes and dietary fiber preparations.

### 2.5 Control interventions

Interventions may include: No treatment, The placebo, Non-drug interventions (eg, diet, exercise, etc), Conventional western medicine hypoglycemic drugs (eg, metformin, euglycemic, etc), Insulin (any kind of insulin). Combined interventions are allowed as long as all groups in the randomized trial receive the same combined intervention.

### 2.6 Types of outcome measures

#### 2.6.1 Main outcomes

1. Glycosylated hemoglobin A1c;
2. fasting blood-glucose;
3. Two Hours Postprandial Blood Glucose (2hPBG).
4. Fasting insulin
5. Two Hours Postprandial insulin
6. Homeostasis model assessment of insulin resistance,

#### 2.6.2 Additional outcomes

1. Low Density Lipoprotein (LDL)
2. High Density Lipoprotein (HDL)
3. triglycerides (TG)
4. total serum cholesterol (TC).

## 3 Data sources

### 3.1 Electronic searches

The following data bases will be searched to identify eligible studies: PubMed, Embase, Cochrane Library, Web of Science, Nature, Science on line, Chinese Biomedical Database WanFang, VIP medicine information, and China National Knowledge Infrastructure. The time range is: the starting time is determined according to the first literature available, and the deadline is August 2020.

### 3.2 Other search resources

**Figure 1:**
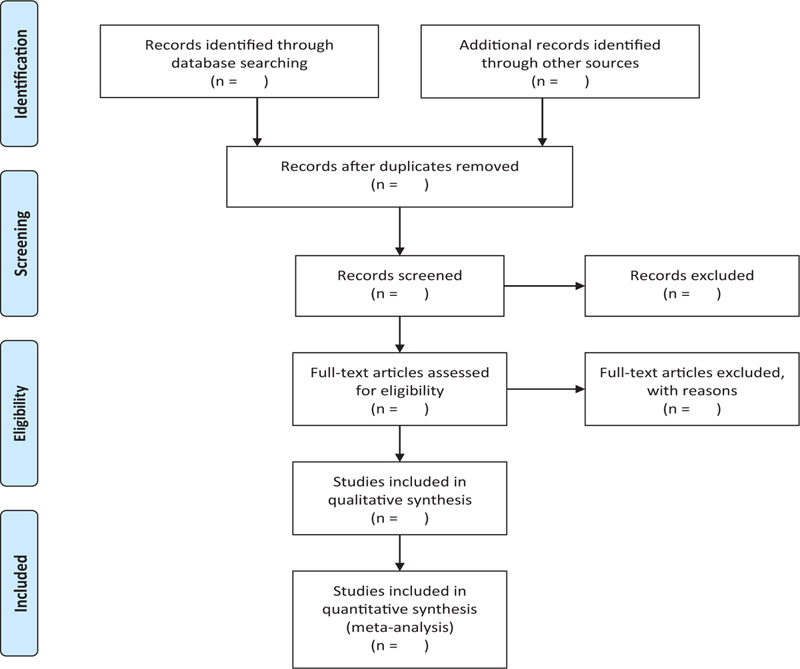
The research flowchart. This figure shows the identification, screening, eligibility and included when we searching articles.

In order to get more complete evidence, we will also retrieve other related documents by manually, such as medical textbooks, clinical laboratory manuals and so on. If it is necessary we will contact with trail author to obtain the latest clinical data. Moreover, studies associated with the review will be identified via evaluating related conference proceedings. The research flowchart is shown in Figure 1.

### 3.3 Search strategy

The following search terms will be used: randomized controlled trial/RCT; gestational hyperinsulinism/gestational Compensatory Hyperinsulinemia/Endogenous Hyperinsulinism in pregnancy Multiple dietary fiber/Fibers/Wheat Bran/ dietary fiber (DF). different retrieval strategies in Chinese and foreign databases will be used. Language restrictions are Chinese and English. There is no publication restriction. Here we take the search strategy in PubMed as an example and list in Table 1.

**Table 1:**
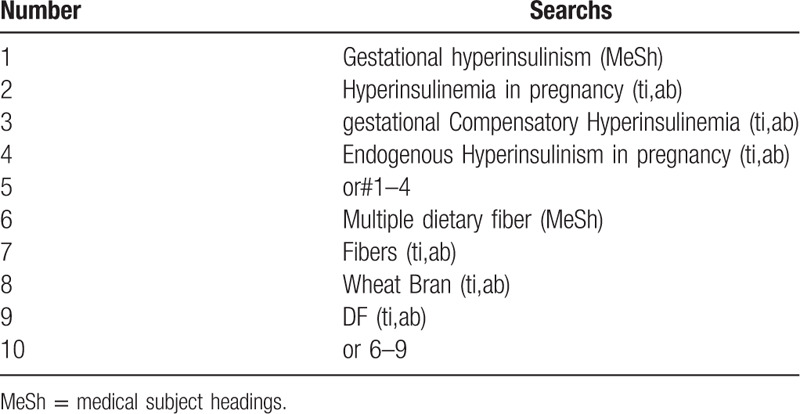
The research strategy. Table 1 Search stragtegy sample of PubMed.

## 4 Data collection and analysis

### 4.1 Study selection

All articles in the search results were independently evaluated by 2 independent researchers (BY, MW) according to inclusion and exclusion criteria. Reviewers will then independently extract and collect the data included in the study using pre-designed data collection forms. Discrepancies will be discussed and resolved by consensus with a third author (XXZ).

### 4.2 Data extraction and management

The following information will be extracted from each study.:1)Normal test characteristics: title, author, year. 2)baseline data: sample size, age, gender, diagnostic criteria, course of disease. 3)interventions: multiple dietary fiber diets, control of intervention details, intervention. If the information is not enough, we will contact experts and authors in this field to get relevant information.

### 4.3 Assessment of the reporting quality and risk of bias

The risk of bias will be assessed by 2 independent authors (BY and MW), together with completing the STRICTA checklist.^[9]^ The Cochrane System Evaluator's Manual give the evaluation criteria for authors to evaluated the RCTs’ quality. Assessing the risk of bias:

1. random sequence generation;
2. allocation concealment;
3. blinding of participants and personnel;
4. blinding of outcome assessment;
5. incomplete outcome data;
6. selective outcome reporting;
7. other bias.

Any disagreement will be discussed or consulted with a third reviewer. Each them will be described from 3 levels: “high risk”, “low risk” and “unclear”.

### 4.4 Measures of a treatment effect

The dichotomous outcomes will be expressed by the Odds ratio (ORs), while the continuous data will use the Standardized mean difference (SMD). All these outcomes report 95% confidence intervals.

### 4.5 Management of missing data

We will take the method of contacting corresponding authors to obtain the missing data. If there is no response, we will analyze only the available data and describe the reason and impact of this exclusion in the paper.

### 4.6 Assessment of a reporting bias

The bias of publication will be explored through funnel plot analysis. If the funnel plot show asymmetry, it will be evaluated via the Egger and Beg tests, and *P* value < .05 means the publication bias is significant.

### 4.7 Assessment of heterogeneity

There are 2 main methods for testing heterogeneity, namely graphical method (funnel plot, forest plot) and statistical test (*Q* value statistic test, *I* ^2^ statistic test, *H* statistic test). The *I* ^2^ statistic test method shows us When *I* ^2^ is 0, it means that studies are completely homogeneous, If *I* ^2^ > 50%, it indicates there is heterogeneity in studies.

### 4.8 Data synthesis and grading of quality of evidence

The results of the study will be analyzed by RevMan 5.0 software provided by Cochrane collaborate on network. The binary data will be expressed by the odds ratio, while the continuous data will use the mean difference (MD). To test the heterogeneity of the research results, when the *I* ^2^ < 50% or *P* > .1, the heterogeneity is significant. The random effect model was used for the meta-analysis, otherwise, we choose the fixed effect model.

### 4.9 Subgroup analysis

???

### 4.10 Sensitivity analysis

Sensitivity analysis can not only assess the stability and reliability of the conclusions of the Meta-analysis, but also assess whether the changes in the results are related to the impact of a single study. If the stability of the conclusion is poor, we can achieve When the heterogeneity test results are heterogeneous, we need to clarify the source of the heterogeneity by subgroup analysis. The effects of different types of therapy including design scheme, severity of illness, age, sex, and mild or severe T2DM were analyzed. We will also delete low-quality and/or medium-quality studies to check the robustness of the results.

The purpose of increasing stability by changing the analysis model, inclusion and exclusion criteria, or excluding a certain type of literature.

### 4.11 Ethics and dissemination

We will publish the system review results in peer-reviewed journals, disseminated in meetings or in peer-reviewed publications. Aggregated published data will be used to exclude data of individuals, so there is no need for obtaining the ethical approval or patients’ informed consent.

## 5 Discussion

Dietary fiber is the general term for polysaccharide carbohydrates that are not digested by human digestive enzymes. It is called the “seventh nutrient”, including soluble DF and insoluble DF. Multiple DF therapy can be used for a variety of metabolic diseases, such as obesity, hyperlipidemia, type 2 diabetes and so on. Control blood sugar through diet intervention in recent years has become a hot research, dietary management can help you to target and reduce blood glucose fluctuations.^[7]^ And the existing research confirmed that eating Multiple DF can effectively improve the insulin resistance in patients with type 2 diabetes, increase insulin sensitivity,^[8]^ for gestational diabetes mellitus research has also confirmed that patients with a reasonable diet to control blood sugar have obvious positive effect,^[10]^ and dietary food most in patients has good practicability and easy to implement, easy to promote.

The efficacy of Multiple DF in the treatment of hyperinsulinemia in pregnancy has been confirmed from the following aspects:

(1) Reducing blood glucose and Gl-ycosylatedhemoglobin.^[11]^
(2) increase insulin sensitivity,[^12^,^13^]
(3) improve intestinal flora imbalance and increase beneficial flora.
(4) Promote islet function repair.^[14]^
(5) improve lipid metabolism disorder.
(6) Regulate immunity.

The improvement of gestational hyperinsulinemia by Multiple DF diets may be mainly related to the characteristics of DF and intestinal flora.

The viscosity of DF can delay gastric emptying, and slow down the absorption of nutrients and starch in the small intestine, and its water retention can increase the volume of chyme in the gastrointestinal tract, causing a sense of satiety. However, insoluble DF can accelerate the transport speed of food in the digestive tube, thus rapidly producing PYY3–36.^[15]^ Pyy3–36 is a gastrointestinal hormone that suppresses appetite and binds to receptors in the hypothalamus to produce a sense of satiety.^[16]^ Insoluble DF also absorbs toxic substances in food to prevent constipation and diverticulitis,

Intestinal flora affects the metabolism and physiological function of the host in many ways, and it has become a research hotspot at home and abroad to improve the occurrence and development of chronic diseases by regulating the structure and diversity of intestinal flora. The imbalance of intestinal flora is closely related to the occurrence and development of metabolic diseases, such as obesity and polycystic ovary syndrome.[^16^,^17^,^18^] DF therapy can influence the development of various diseases by regulating intestinal flora and its metabolites (such as short-chain fatty acids).

DF can be fermented by intestinal flora to produce short-chain fatty acids, mainly including acetic acid, propionic acid and butyric acid. Because the gut contains a variety of bacteria that produce short-chain fatty acids, each of which has a different substrate requirement. Thus, in the same amount of time, giving more DF may satisfy the metabolic requirements of more bacteria and produce more short-chain fatty acids. Short-chain fatty acids can strengthen the intestinal mucosal barrier, reduce insulin resistance caused by endotoxin entering the blood,[^19^,^20^] and slow down hyperinsulinemia in pregnancy. Short-chain fatty acids also downregulate peroxidase proliferator-activated receptor to activate amp-activated protein kinase pathways, promote fatty acid oxidation and energy expenditure, and reduce body weight.^[20]^

At the same time, multi-DF diet can promote the growth of beneficial bacteria such as lactobacillus and improve the intestinal environment, thus improving insulin resistance and reducing hyperinsulinemia during pregnancy. Zhang et al found that ribonucleic acid in exosomes of ginger promoted the reproduction of Lactobacillus by changing the gene expression of Lactobacillus and induced the production of more aromatics receptor ligands, thus improving the barrier function of intestinal tract.^[21]^

In conclusion, systematic review and meta-analysis are helpful to determine the potential value of multiple dietary fibers in the treatment of hyperinsulinemia in pregnancy. This study can provide a new treatment idea for the current clinical hyperinsulinemia pregnant women, so as to provide better clinical services and improve the fertility and fertility rate.

## Author contributions

**Conceptualization**: Botong Yang, Mengzhu Wu, Xinxia Zhang, Lizhen Wang.

**Data curation**: Botong Yang, Mengzhu Wu.

**Formal analysis**: Botong Yang, Jia Xia.

**Methodology**: Botong Yang, Mengzhu Wu, Xinxia Zhang, Lizhen Wang.

**Project administration**: Xinxia Zhang.

**Resources**: Botong Yang, Mengzhu Wu, Jia Xia.

**Software**: Botong Yang, Mengzhu Wu.

**Supervision**: Xinxia Zhang.

**Writing – original draft**: Botong Yang

**Writing – review & editing**: Xinxia Zhang.
